# An audit of the management of ectopic pregnancies in a district hospital, Gauteng, South Africa

**DOI:** 10.4102/phcfm.v10i1.1757

**Published:** 2018-10-30

**Authors:** Doudou K. Nzaumvila, Indiran Govender, Gboyega A. Ogunbanjo

**Affiliations:** 1 Department of Family Medicine and Primary Health Care, Sefako Makgatho Health Sciences University, South Africa; 2 Dr George Mukhari Academic Hospital, Ga-Rankuwa, South Africa

## Abstract

**Background:**

Ectopic pregnancy (EP) is a life-threatening condition; at Odi Hospital, approximately 7–10 EPs are managed weekly. Our study is the first to assess the management of this life-threatening condition at Odi Hospital.

**Aim:**

This study aimed to determine the incidence of EP and to assess the profile of women who presented with EP at Odi District Hospital from 01 January 2010 to 31 December 2014.

**Setting:**

The study was conducted at Odi District Hospital, located in Mabopane, a township in Gauteng province, 45 km north of Tshwane, South Africa (SA).

**Methods:**

This was a cross-sectional study.

**Results:**

We analysed 263 completed patient records. The incidence rate was 22 per 1000 live births. The mean age was 28.9 years (SD ± 6.09), 57% were within the age group of 25–34 years, 90.9% were single and 85.2% were unemployed. Abdominal pain was the most common presenting complaint (81.1%). Ninety-nine (37.8%) were in a state of haemorrhagic shock. Possible risk factors were not documented in the patient files for 95%. A third (34.2%) were operated on within 4 hours of consultation. Early management was associated with poor record-keeping (*p* = 0.02). There was a delay in confirming the diagnosis in 48.7%. It was associated with gestational age (*p* = 0.0017), previous abdominal surgery (*p* = 0.0026), normal haemoglobin level at the time of consultation (*p* = 0.0024), considerable haemoperitoneum at operation (*p* < 0.00001) and per vaginal bleeding (*p* = 0.003).

**Conclusion:**

The study highlighted the need to emphasise the importance of good record-keeping and documentation in patients, as well as the urgent need for ultrasound skills training among clinicians to implement the Essential Steps in Managing Obstetric Emergencies programme at this hospital to improve the management of EP and other obstetric emergencies.

## Introduction

Ectopic pregnancy (EP) is a complication of pregnancy characterised by an abnormal implantation of the fertilised ovum in tissues other than the endometrial lining of the uterus.^1,2^ In 97% of cases, the EP is located in the fallopian tube, and sometimes the EP occurs in the pelvic or abdominal cavity. Very rarely does it occur in the cervix, and less-reported sites are the ovaries and broad ligaments.^3,4^ This abnormal localisation of pregnancy is a life-threatening obstetrical condition found in women of reproductive age, and it is a leading cause of maternal death when rupture occurs in the first trimester of pregnancy.^5,6,7,8^ The aetiology of EP is not well understood, but risk factors associated with EP include pelvic inflammatory disease, puerperal sepsis, previous termination of pregnancy, post-abortion sepsis, appendicitis and use of intrauterine contraceptive devices.^5,8,9^ The incidence of ectopic pregnancies varies between studies and from one country to another. Globally, the incidence ranges from 1 EP per 28 live births to 1 EP per 106 live births.^9^ In developed countries, the incidence is reported to be 0.67% and very stable. The early diagnosis of this condition allows for definitive medical treatment in developed countries with very few surgical treatments.^10^ Various African studies have reported an incidence ranging between 1.2% and 2.7%.^6,11,12,13,14,15^ There has been a rise in the incidence of EP in many African countries in the past few years.^8,12,16^ Etuknwa et al.^6^ indicated that the incidence of EP has tripled over the last 30 years. The current standard of treatment in Africa, as a resource-poor region, is emergency laparotomy with salpingectomy of the involved fallopian tube. The medical treatment of EP is still suboptimal and involves the use of methotrexate. EP can be diagnosed at an early stage and optimally treated cost-effectively, with medical treatment as outpatient or laparoscopically, with no need for major surgery.^16,17^

In South Africa (SA), EP was reported to occur in 11 out of every 1000 reported pregnancies, and the mortality rate was estimated at 2.0%.^18^

Many patients are 26–28 years of age and multigravida. The main complaints are either bleeding or a combination of bleeding and abdominal pain.^16^ Most women with this condition present with ruptured or bleeding tubal pregnancies, mainly because of poor contraception services, high numbers of unintended pregnancies and few early pregnancy confirmation visits.^17^ Emergency laparotomy is the main treatment, followed by emergency laparoscopy and very little medical treatment.^16,17^

No previous study on EP has taken place at Odi District Hospital. This study aimed to determine the incidence of EP and assess the profile of women who presented with EP at Odi District Hospital from 01 January 2010 to 31 December 2014.

## Research methods and design

### Study design

This was a cross-sectional, retrospective study.

### Setting

The study was conducted at Odi District Hospital, located in Mabopane, a township in Gauteng province, 45 km north of Tshwane, SA. The hospital is a 198-bed district hospital that serves a population of 524 632 people from Gauteng province and 355 905 people from North-West province. The hospital has a large referral network of numerous clinics from both provinces. Odi Hospital employs two specialists (family medicine), 20–22 medical officers and one to four clinical associates. It serves as a medical teaching platform for the Sefako Makgatho Health Sciences University, Ga-Rankuwa. Family medicine registrars (five to seven) rotate in different departments, whereas one senior registrar from the related departments of Dr Georges Mukhari Academic Hospital, Ga-Rankuwa, supports certain departments, such as obstetrics and gynaecology and internal medicine (on a daily and weekly basis, respectively).

The study population consisted of all EP patients managed from 01 January 2010 to 31 December 2014, which was 338 EP patients operated on during the study period.

### Data collection

We included all the cases of EP operated on at Odi Hospital during the study period and excluded all transfers, as we could not acquire feedback once patients were discharged to their local clinics. Operating theatre records were reviewed for files of ectopic pregnancies operated on during the study period. A list of files was submitted to the registry department to retrieve the files. All files with adequate records were included in the study. From the theatre register, 338 records of ectopic pregnancies were obtained, of which 276 files were available to the researchers for data collection. Thirteen files were inadequate because of poor records or patients having been transferred to other hospitals because of intubation failure, which yielded 263 files. A data collection sheet was developed and used to extract the required information from the medical files of patients operated on for EP. Maternal and obstetric baseline characteristics included gestational age, number of previous pregnancies, last normal menstrual periods (LNMP) date, family planning obstetrical and relevant surgical history, previous sexually transmitted infections (STIs), complaints at the time of presentation, medical and clinical findings, laboratory investigations, ultrasound scan findings and post-operative data, blood transfusions and any complications. To ensure the validity and reliability of data, a pilot study was conducted by two independent researchers in March 2017 using 22 files of admitted patients with EP, in order to test the data collection sheet. All comments and feedback regarding this pilot study were considered to finalise the data collection sheet.

### Data analysis

The researchers used Microsoft Excel^TM^ 2010 software to capture data and import it into Instat^TM^ software, version 97, for analysis. Data were analysed using descriptive statistics. Bivariate analyses were performed using the Fisher exact test for association of variables.

Statistical significance of results was set at *p* ≤ 0.05.

### Results

From the expected 338 files, only 276 were retrieved, and 13 were unsuitable because of incomplete data. Therefore, 263 (77.8%) files were analysed for this study.

### The incidence of ectopic pregnancy

Over the five-year period, the hospital recorded 15 281 deliveries. The incidence rate of EP in this study was 22 per 1000 births.

### Baseline characteristics

The mean (SD) age of women was 28.9 years (SD ± 6.09), with an age range of 17–46 years. Approximately two-thirds of the patients with EP were aged between 25 and 34 years. The majority were single (90.9%) and unemployed (85.2%) (Table 1).

**TABLE 1:** Baseline characteristics of patients with ectopic pregnancy.

Characteristics	2010		2011		2012		2013		2014		Total	
	*N*	%	*N*	%	*N*	%	*N*	%	*N*	%	*N*	%
**Age groups**												
15–24	12	30.8	11	22.0	13	17.1	11	22.0	17	35.4	64	24.3
25–34	20	51.3	29	58.0	52	68.4	27	54.0	22	45.8	150	57.0
35 and above	7	17.9	10	20.0	11	14.5	12	24.0	9	18.8	49	18.7
**Marital status**												
Single	32	82.1	47	94.0	74	97.4	40	80.0	46	95.8	239	90.9
Married	5	12.8	2	4.0	1	1.3	6	12.0	2	4.2	16	6.1
Not documented	2	5.1	1	2.0	1	1.3	4	8.0	0		8	3.0
**Occupation**												
Employed	3	7.7	2	4.0	7	9.2	1	2.0	3	6.2	16	6.1
Student	8	20.5	1	2.0	3	3.9	2	4.0	1	2.1	15	5.7
Unemployed	26	66.7	46	92.0	65	85.5	43	86.0	44	91.7	224	85.2
Not documented	2	5.1	1	2.0	1	1.3	4	8.0	0	0.0	8	3.0
**Total**	**39**	**14.8**	**50**	**19.0**	**76**	**28.9**	**50**	**19.0**	**48**	**18.3**	**263**	**100.0**

### Gynaecological profile

EPs were more frequent in multigravida (46%). Slightly over one-fifth (22.4%) presented within four weeks of their LNMP. In addition, the audited files showed that in 42.2%, the doctors’ notes did not have any record of previous gynaecological history. For previous surgical history and STIs, there was no data in 96.6% and 95.4% of patients, respectively (Table 2).

**TABLE 2:** Gynaecological profile of patients.

Characteristics	2010		2011		2012		2013		2014		Total	
	*N*	%	*N*	%	*N*	%	*N*	%	*N*	%	*N*	%
**Gynecological profile**												
Primigravida	3	7.7	6	12.0	10	13.16	8	16.0	4	8.33	31	11.8
Multigravida	18	46.1	25	50.0	33	43.42	29	58.0	14	25.34	121	46.0
Not documented	18	46.1	19	38.0	33	43.42	13	26.0	28	58.33	111	42.2
**Gestational age as per LNMP**												
Within 4 weeks	10	25.6	13	26.0	15	19.70	10	20.0	11	22.90	59	22.4
5–8 weeks	4	10.3	7	14.0	12	15.80	11	22.0	14	29.20	48	18.3
9 and more	2	5.1	4	8.0	5	6.60	0	0.0	3	6.20	14	5.3
Not documented	23	59.0	26	52.0	44	57.90	29	58.0	20	41.70	142	54.0
**Previous surgical history**												
Previous abdominal surgery	0	0.0	3	6.0	0	0.00	1	2.0	1	2.10	5	1.9
Previous EP	1	2.6	0	0.0	1	1.30	2	4.0	0	0.00	4	1.5
Not documented	38	97.4	47	94.0	75	98.70	47	94.0	47	97.9	254	96.6
**Previous STI**												
No previous STI	1	2.6	3	6.0	5	6.60	1	2.0	0	0.00	10	3.8
Previous STI	1	2.6	0	0.0	1	1.30	0	0.0	0	0.00	2	0.8
Not documented	37	94.8	47	94.0	70	92.10	49	98.0	48	100.00	251	95.4
**Total**	**39**	**14.8**	**50**	**19.0**	**76**	**28.90**	**50**	**19.0**	**48**	**18.30**	**263**	**100.0**

LNMP, last normal menstrual period; EP, ectopic pregnancy; STI, sexually transmitted infection.

### Main complaints at presentation

Abdominal pain was the main complaint of patients, accounting for 81.1% of the recorded symptoms. There were complaints of non-gynaecological symptoms, such as syncope or collapse, in 12.6% of patients. More than one-third of patients had signs of shock at the time of presentation to the hospital (Table 3).

**TABLE 3:** Main complaints, investigation results and missed diagnosis for ectopic pregnancy.

Characteristics	2010		2011		2012		2013		2014		Total	
	*N*	%	*N*	%	*N*	%	*N*	%	*N*	%	*N*	%
**Complaint**												
Abdominal pain	33	84.6	47	94.0	56	73.7	43	86.0	41	85.40	216	81.1
Amenorrhea	7	17.9	2	4.0	11	14.5	2	4.0	4	8.30	26	9.9
Weakness	3	7.7	4	8.0	1	2.6	1	2.0	3	6.20	12	4.6
Dizziness	8	20.5	2	4.0	5	6.6	2	4.0	6	12.50	23	8.7
PV bleeding	24	61.5	25	50.0	42	55.3	27	54.0	21	21.70	139	52.9
Others	5	12.8	2	4.0	8	10.5	8	16.0	9	18.70	33	12.6
**Clinical presentation**												
Shock	5	12.8	17	34.0	12	15.8	11	22.0	18	37.50	63	24.0
Not in shock	34	87.2	33	66.0	64	84.2	39	78.0	30	62.50	200	76.0
**Investigation**												
Ultrasound	33	84.6	45	90.0	68	89.5	46	92.0	44	91.70	236	89.7
Culdocentesis	0	0.0	1	2.0	1	1.3	0	0.0	0	0.00	2	0.7
Quantitative βHCG	11	19.1	7	14.0	12	15.8	8	16.0	3	6.20	41	15.6
Missed EP for other diagnosis	25	19.5	18	14.1	38	29.7	31	24.2	16	12.50	128	100.0
Incomplete abortion	5	20.0	3	25.0	2	5.3	1	3.2	2	12.50	13	10.2
Threatened abortion	14	56.0	10	38.0	19	50.0	16	51.6	8	50.00	67	52.3
UTI	3	12.0	4	22.2	12	31.5	11	34.5	1	6.25	31	24.2
Other	3	12.0	2	11.1	5	13.2	3	9.7	5	31.25	17	13.3
**Total**	**39**	**14.8**	**50**	**19.0**	**76**	**28.9**	**50**	**19.0**	**48**	**18.20**	**-**	**-**

UTI, urinary tract infection; βHCG, beta-human chorionic gonadotropin; EP, ectopic pregnancy.

### Investigations

The mean haemoglobin (SD) was 9.7g/dL (± 2.5). Ultrasound examination was performed in 236 patients (89.7%) but the diagnosis was missed in 117 (44.9%). A quantitative beta-human chorionic gonadotropin (HCG) test was performed in 41 patients (15.6%) and culdocentesis in three patients (0.7%). In total, 128 patients (48.7%) were misdiagnosed. Among them were patients with per vaginal (PV) bleeding diagnosed initially as threatened abortion 67 (52.3%) (see Table 3).

### Operative findings

The delay between presentation at the hospital and laparotomy ranged from within the first hour of consultation to 336 h. In 34.2%, patients were operated on within the first 4 h. Ruptured EP accounted for 74.5% of cases. The commonest site of EP was the fallopian tube (59.7%). Blood transfusions were required in 79.5% of cases, with 41.8% receiving three or more units of blood. The mean operative time for the laparotomy was 60.2 min (± 33.7) and 40.3% were completed within 1 h. The average stay in hospital was 2–4 days. Few post-operative complications were reported, with one bladder injury (0.38%) and two patients with excessive haemorrhage being transferred post-operatively (0.76%) (Table 4). The mean (SD) volume of the haemoperitoneum was 674.9 mL (± 53.5).

**TABLE 4:** Perioperative findings, complications and hospital stay.

Characteristics	2010		2011		2012		2013		2014		Total	
	*N*	%	*N*	%	*N*	%	*N*	%	*N*	%	*N*	%
**Time between consultation and operation (hours)**												
0–4	9	23.1	19	38.0	31	40.8	15	30.0	16	33.3	90	34.2
5–9	9	23.1	18	36.0	21	27.6	11	22.0	24	50.0	83	31.6
10–14	9	23.1	3	6.0	5	6.6	6	12.0	3	6.25	26	9.9
15–19	5	12.8	1	2.0	0	0.0	4	8.0	0	0.0	10	3.8
20–24	3	7.7	2	4.0	5	6.6	5	10.0	0	0.0	15	5.7
25–48	4	10.3	1	2.0	7	9.2	6	12.0	3	6.2	21	8.0
49–96	0	0.0	4	8.0	4	5.3	3	6.0	2	4.2	13	4.9
97 or more	0	0.0	2	4.0	3	3.9	3	6.0	0	0.0	5	1.9
**Site**												
Abdominal	0	0.0	1	2.0	1	1.3	0	0.0	2	4.2	4	1.5
Ampulla	0	0.0	8	16.0	10	13.1	2	4.0	4	8.3	24	9.1
Infundibulum	4	10.3	7	14.0	6	7.9	4	8.0	9	18.7	30	11.4
Isthmus	2	5.1	2	4.0	3	3.9	1	2.0	0	0.0	8	3.0
Fallopian (no precision of site)	27	69.2	30	60.0	43	56.6	32	64.0	25	52.1	157	59.7
Other localisation	0	0.0	0	0.0	3	3.9	0	0.0	0	0.0	3	1.1
Not documented	6	15.4	11	22.0	10	13.2	11	22.0	8	16.7	37	14.1
**Course of ectopic**												
Ruptured	32	82.1	31	62.0	63	82.9	34	68.0	36	75.0	196	74.5
Not ruptured	7	17.9	17	34.0	7	9.2	9	18.0	6	12.5	46	17.5
Not documented	0		2	4.0	6	7.9	7	14.0	6	12.5	21	8.0
**Blood transfusion (in units)**												
Not transfused	15	38.5	7	14.0	21	27.6	8	16.0	3	6.3	54	20.5
1	10	25.6	6	12.0	6	15.4	5	10.0	4	8.3	31	11.8
2	7	18.0	11	22.0	25	32.9	10	20.0	15	31.3	68	25.9
3 or more	7	18.0	26	32.0	24	31.6	27	54.0	26	54.2	110	41.8
**Operating time (in minutes)**												
Less than 30	4	10.3	2	4.0	4	5.3	3	6.0	3	6.3	16	6.1
30–59	22	56.4	19	38.0	31	40.8	14	40.0	20	41.7	106	40.3
60–89	7	18.0	13	26.0	25	32.9	14	24.0	12	25.0	71	27.0
90 or more	2	5.1	5	10.0	4	5.3	3	6.0	10	20.8	24	9.1
Not documented	4	10.3	11	22.0	12	15.8	16	32.0	3	6.3	46	17.5
Complications	2	5.1	0	0.0	1	1.3	0	0.0	0	0.0	3	1.1
**Hospital stay (in days)**												
2–4	32	82.1	36	72.0	57	75.0	38	76.0	40	83.3	203	77.2
5–7	7	18.0	13	26.0	15	19.7	11	22.0	5	10.4	51	19.4
8 or more	0	0.0	1	2.0	4	5.2	1	2.0	3	5.3	9	3.4
**Total**	**39**	**14.8**	**50**	**19.0**	**76**	**28.9**	**50**	**19.0**	**48**	**18.2**	**263**	**100.0**

Sixty-eight patients (37.9%) had circulatory shock at the time of consultation. A normal haemoglobin blood level at the time of consultation was associated with delay in the confirmation of the diagnosis of EP and laparotomy (*p* = 0.0024), gestational age within the first 4 weeks of LNMP (*p* = 0.0017), previous abdominal surgery (*p* = 0.0026), PV bleeding (*p* = 0.003) and patients who were haemodynamically stable (*p* = 0.0019). There was also a statistically significant association between patients with no gynaecological profile documented and early laparotomy (*p* = 0.002) (see Table 5).

**TABLE 5:** Sub-analysis of delayed laparotomy.

Characteristics	Delayed laparotomy		No delayed laparotomy		*odd ratio*	Confidence interval	*p*
	*N*	%	*N*	%			
**Age**	63	49.20	76	56.30	0.75	0.46–1.22	0.27000
**Marital status**							
Single	114	89.10	125	92.59	0.65	0.28–1.52	0.39000
Married	6	4.69	10	7.41	0.54	0.19–1.56	0.30000
Not documented	4	3.13	4	2.96	0.94	0.23–3.90	1.00000
**Occupation**							
Employed	8	6.25	8	5.92	0.95	0.30–2.60	1.00000
Student	6	4.69	9	6.67	0.61	0.20–1.80	0.43000
Unemployed	107	83.59	117	86.67	0.78	0.40–1.50	0.49000
No documented	4	4.13	4	2.96	0.94	0.23–3.90	1.00000
**Gynaecological profile**							
Primigravida	18	6.72	13	9.63	1.53	0.72–3.30	0.34000
Multigravida	65	5.78	56	41.48	1.40	0.89–2.40	0.14000
Not documented	45	35.16	66	48.89	0.57	0.34–0.90	0.02000
**Gestational age**							
Within 4 weeks	37	28.91	22	16.30	2.10	1.51–3.79	0.00170
5–8 weeks	22	17.19	26	12.26	0.87	0.46–1.63	0.75000
9 weeks and more	5	3.91	9	6.67	0.57	0.18–1.75	0.41000
Not documented	64	50.00	78	57.78	0.73	0.45–1.20	0.22000
**Previous surgery**							
Previous abdominal surgery	5	3.91	0		10.83	0.59–198.04	0.00260
Previous EP	2	1.56	2	1.48	0.94	0.13–6.80	1.00000
Not documented	121	94.53	133	98.51	0.26	0.05–1.28	0.09000
**Previous STI**							
Number of previous STI	6	4.69	4	2.96	1.61	0.44–5.84	0.53000
Previous STI	2	1.56	0		5.35	0.25–112.72	0.23000
Not documented	120	93.75	131	97.04	0.46	0.13–1.56	0.24000
**Complaints**	97	75.78	119	88.15	0.42	0.22–0.81	0.01000
Abdominal pain	17	13.28	9	6.67	2.14	0.91–5.00	0.09000
Amenorrhea	3	2.34	9	6.67	0.34	0.09–1.27	0.14000
Weakness	80	62.50	59	43.70	2.15	1.31–3.52	0.00300
PV bleeding	23	17.97	14	10.37	1.89	0.93–3.88	0.11000
Dizziness	17	13.28	16	11.85	0.80	0.39–1.68	0.58000
**Clinical presentation**							
Not in shock	46	35.94	22	16.30	2.50	1.40–4.45	0.00190
**Investigations**							
Number of u/s examination	10	7.81	16	11.85	0.63	0.27–1.45	0.30000
U/s examinations done	118	92.19	118	87.4	1.7	0.75-3.87	0.30000
Culdocentesis	1	0.78	1	0.74	1.05	0.06–17.00	0.17000
Quantitative βHCG	24	8.96	17	12.59	1.60	0.81–3.14	0.17000
Normal Hb	33	25.78	15	11.11	2.80	1.43–5.42	0.00240
**Perioperative findings**							
Ruptured	99	77.34	97	71.85	1.34	0.76–2.34	0.32000
Not ruptured	20	15.63	26	19.26	0.78	0.40–1.50	0.52000
Not documented	9	7.03	12	8.89	0.77	0.40–1.47	0.65000
Large haemoperitoneum	43	33.59	14	10.37	4.37	2.51–8.50	< 0.00001
**Total**	**128**	**48.67**	**135**	**51.33**	**-**	**-**	**-**

UTI, urinary tract infection; EP, ectopic pregnancy; U/s, ultrasound; STI, sexually transmitted infection; PV, per vaginal; βHCG, beta-human chorionic gonadotropin; Hb, haemoglobin.

## Discussion

The present study recorded an incidence of EP of 2.2%, which is in line with other African studies. In 2009, Musa et al. reported an incidence of 1.74%, and in 2016 incidences of 1.79% and 1.8% were reported by Murugesan et al. and Prasanna et al., respectively.^15,19^ However, the last two cited studies were conducted in tertiary hospitals, where more complicated cases are managed, whereas this study was conducted in a district hospital, dealing mainly with primary-level cases. The current study found an incidence rate higher than that reported by Amoko et al. in 1995 at the Umtata General Hospital, SA (1.1%).^16^ This suggests that there is an increase in EP, as reported by previous non-South African studies.^6,15^ Among the risk factors that may explain the rising incidence of EP are pelvic inflammatory disease, intrauterine contraceptive devices and tubal surgery.^5,8,9^ A South African study reported up to 86% of the cases of EPs had evidence of previous pelvic infection, thus making pelvic inflammatory disease the most important risk factor for EP.^16^ This association couldn’t be established in our study as such information was missing in many of the patients’ files.

The findings could have been higher than 22 per 1000 live births, as other possible cases of EP were not sent to the theatre, where the researchers collected the patients’ records. As a district hospital (Level 1), Odi Hospital, which has a list of criteria for transferring patients to a higher level of care, transferred some patients who met these criteria. This includes patients with a haemoglobin level of 4 g/l and less; secondly, patients with comorbidities whose condition may have been complicated with anaesthesia, such as severe pneumonia; and finally, a lack of anaesthetic skills among the doctors to deal with complicated or haemodynamically unstable patients. It is common practice that some doctors at this hospital booked for an anaesthetic call preferred to transfer patients to other hospitals, when they did not feel comfortable with general anaesthesia. This is mostly the case with community service doctors and, to a certain extent, junior medical officers. Ideally, there should be three doctors in theatre during an operation, with the most skilled doctor responsible for anaesthesia. However, at the time of the study, there was a shortage of skilled doctors, which resulted in patients being unnecessarily transferred to other hospitals, usually the closest tertiary hospital (Dr. George Mukhari Academic Hospital). The Essential Steps in Managing Obstetric Emergencies (ESMOE) programme, which has been developed to improve the skills of doctors in managing obstetric emergencies, is highly relevant in this district health setting, especially as it includes a section on improving anaesthetic care for pregnant patients requiring invasive procedures.

The baseline characteristics showed a mean age of 28.96 years (SD ±6.09). The age group 25–34 years (57%) had the highest frequency of EP for 10-year age group bands and the age group 21–25 years had the highest frequency of 32% for 5-year age group bands. In comparison, a study with a sample size (298 cases) closer to ours, which had 5-year age group bands, found the highest incidence of 36% among the group age of 26–30 years, which is in keeping with our findings.^7^ However, this finding contrasted with the 60% in the age group of 21–25 years by Abdul, who had a sample size of 278 cases of EP.^10^ The incidence of EP may have other factors than age groups to explain the differences.

Although possible correlations do not imply causality, we found that most patients treated for EP during the study period were single (90.9%), which corroborates the findings of Etuknwa et al., albeit they found a lower percentage of 51.4%. However, other studies reported a preponderance of married women with EP.^8,11^ Other authors have attributed the high incidence of EP among single women to the fact that they are more prone to STIs because of possible multiple sexual partners, and that they usually resort to an induced abortion (which is often performed by non-qualified personnel, resulting in complications and late presentation of unwanted pregnancies).^12^ This may also be relevant in the South African context; despite the fact that the country’s legislation and medical facilities are made convenient for patients to have access to services offering free medical termination of pregnancy, there are still patients who are not using the opportunity to terminate pregnancy and prefer backdoor abortions for many reasons.^20,21^

Many patients had a low socio-economic status, with 224 (85.2%) being unemployed. This is higher than the unemployment rate in SA reported by Stats South Africa (27.7%).^22^ Given that Odi District Hospital is in a township and offers free healthcare to patients, this figure is not surprising. This concurs with the findings of Murugesan et al., who argued that women with low socio-economic status tend to have poor personal hygiene and poor immunity, which predisposes them to pelvic inflammatory diseases.^8^

Our study showed that 40.7% of patients presented within eight weeks of their LMP, while 54% of the files retrieved did not have records of their LMP. In addition, this study found that EP occurred more in multigravida (46.0%), which corroborates the findings of other studies.^8,17^ However, it differs from the findings of other studies, where most EPs were found among primigravida.^6,15^ This study showed that diagnostic confirmation and laparotomy was delayed in 48.7%. However, most patients with no documented gynaecological records (gravidity, parity) were spared and were operated within 4 hours of presentation to the hospital (*p* = 0.0026).

Most of the patients (81.1%) reported abdominal pain as the main complaint at the time of presentation, which is in line with other studies.^4,6,8^ Vaginal bleeding was reported in 52.9% of the cases, making it the second most common reason for consultation. Similarly, vaginal bleeding was mentioned in many studies, whereas other studies found that pain and amenorrhea were the main symptoms.^4,7,8,19^

Patients in hypovolemic shock accounted for 37.8%, which is in line with a previous African study.^7^ This differs significantly from the study conducted by Amoko et al., who found that 62.2% of their patients were in hypovolemic shock at the time of presentation.^18^ This discrepancy can be explained by the fact that the latter study was conducted at a tertiary-level hospital, which received more complicated cases than our study, in a primary-level hospital.

It was found that only 34.2% of our patients with EP were operated on within 4 h of presentation, while the others had to wait longer. This can be explained by the differential diagnosis of early EP. Although ultrasound was performed, more than half of the initially missed cases were treated as threatened abortion. This can be attributed to the fact that primary-level hospitals have difficulty in attracting sonographers to work in the district hospitals. One can argue that the use of ultrasound helps in diagnosis. However, during the study period, there were no good quality ultrasound machines for a considerable period and the effectiveness of this tool depends on the operator’s skills and the machine itself. In addition, very few doctors had basic ultrasound training at Odi District Hospital. ESMOE training should be considered as part of the solution to this problem.

At surgery 59.7% of ectopic pregnancies were found in the fallopian tubes, which was in line with other studies.^4,7,11,15^ The majority of these EPs were ruptured, which is similar to the findings of other African studies.^8,16^ South African authors believe that this is mainly because of poor contraception services, high numbers of unintended pregnancies, few early pregnancy confirmation visits, late consultation and a poor means of investigation, in comparison to developed countries.^23^ A significant relationship was found between the haemoglobin blood level at the time of consultation and patient haemodynamically stable with the delay in confirmation of the diagnosis of EP and laparotomy. One can argue that medical doctors will consider other differential diagnoses at the early stages of EP (not ruptured). This is substandard clinical care and is a wastage of scarce resources, as by the time the EP is ruptured, more resources will be needed; as demonstrated in this study, 79.47% patients needed blood transfusion. In our context, a high index of suspicion should be applied to any woman of childbearing age with abdominal pain and positive urine (dipstick) pregnancy test.

## Recommendations

The authors recommend that the medical team of Odi Hospital initiate regular random patient file audits to identify and address the root causes behind the delays in diagnosing EP and to ensure that a standard operating procedure is in place to improve EP record-keeping. To the hospital management, the authors recommend that a good quality ultrasonography machine be purchased and a permanent ultra-sonographer employed who will empower the medical team in terms of expertise and training. The district family medicine team should also play a crucial role in training the medical officers using the national ESMOE training programme.

## Strengths and limitations

The strength of this audit is that it is the second study in South Africa to establish the prevalence of EP after more than 20 years and it is the first study of EP conducted at the primary care level, whereas previous studies were at tertiary care level. Study limitations include the transfer of uncomplicated cases of EP to a higher level of care, missing crucial information such as LMP and risk factors for EP, constituted substandard clinical care, and data was only collected from one site. However, the study findings provide information for further studies on EP at primary care level.

## Conclusion

More efforts should be taken to emphasise the quality of patients’ notes and the need to be pro-active in diagnosing early EP. The study highlights the urgent need for ultrasound skills and machine availability and to implement the ESMOE training programme at this hospital to improve the management of EP and other obstetric emergencies.

## References

[1] Chen X, Chen Z, Cao Z, et al The 100 most cited articles in ectopic pregnancy: A bibliometric analysis. Springer Plus. 2016;5(1):1815 https://doi.org/10.1186/s40064-016-3503-8 27803848 10.1186/s40064-016-3503-8 PMC5069217

[2] Chang J, Elam-Evans LD, Berg CJ, et al Pregnancy-related mortality surveillance – United States, 1991–1999. MMWR Surveill Summ. 2003;52(2):1–8. 12825542

[3] Cai Y, Xiao E, Shang Q, Xiao l Ectopic pregnancy in the liver incidentally diagnosed by imaging: A case report. Exp Ther Med. 2017;14:373–376. https://doi.org/10.3892/etm.2017.4478 28672941 10.3892/etm.2017.4478 PMC5488510

[4] Igwebe AO, Eleje GU, Okpala BC An appraisal of the management of ectopic pregnancy in a Nigerian tertiary Hospital. Ann Med Health Sci Res. 2013;3(2):166–170. https://doi.org/10.4103/2141-9248.113655 23919183 10.4103/2141-9248.113655 PMC3728856

[5] Lawani OL, Anozie OB, Ezeonu PO Ectopic pregnancy: A life-threatening gynecological emergency. Int J Women’s Health. 2013;3(5):515–521. https://doi.org/10.2147/IJWH.S49672 10.2147/IJWH.S49672 PMC3751381 23983494

[6] Etuknwa BT, Azu OO, Peter AL, et al Ectopic pregnancy: A Nigerian urban experience. Korean J Obstet Gynecol. 2012;55(5):309–314. https://doi.org/10.5468/KJOG.2012.55.5.309

[7] Panti A, Ikechukwu NE, lukman OO, Yakubu A, Egondu SC, Tanko BA Ectopic pregnancy at Usmanu Danfodiyo University Teaching Hospital Sokoto: A ten year review. Ann Nigerian Med. 2012;6(2):87–91. https://doi.org/10.4103/0331-3131.108128

[8] Murugesan A, Prabhu K, Muthulakshmi M A retrospective study of ectopic pregnancies in a tertiary care hospital. Int J Reprod Contracept Obstet Gynecol. 2016;5(8):2537–2540. https://doi.org/10.18203/2320-1770.ijrcog20162225

[9] Gharoro EP, Igbafe AA Ectopic pregnancy revisited in Benin City, Nigeria: Analysis of 152 cases. Acta Obstet Gynecol Scand. 2002;81(12):1139–1143. https://doi.org/10.1034/j.1600-0412.2002.811207.x 12519110 10.1034/j.1600-0412.2002.811207.x

[10] Abdul F Ectopic pregnancy in Ilorin: A review of 278 cases. Niger J Med. 2000;9(3):92–96.

[11] Udigwe GO, Umeononihu OS, Mbachu II Ectopic pregnancy: A 5 year review of cases at Nnamdi Azikiwe University Teaching Hospital (NAUTH) Nnewi. Niger Med J. 2010;51:160–163.

[12] Aboyeji AP, Fawole AA, Ijaija MA Trends in ectopic pregnancy in Ilorin, Nigeria. Nigerian J Surg Res. 2002;4:6–11. https://doi.org/10.4314/njsr.v4i1.12163

[13] Airede LR, Ekele BA Ectopic pregnancy in Sokoto, Northern Nigeria. Malawi Med J. 2005;17:14–6. https://doi.org/10.4314/mmj.v17i1.10864 27528991 10.4314/mmj.v17i1.10864 PMC3346042

[14] Anorlu RI, Oluwole A, Abudu OO, Adebayo S Risk factors for ectopic pregnancy in Lagos Nigeria. Acta Obstet Gynecol Scand. 2005;84:184–188. https://doi.org/10.1111/j.0001-6349.2005.00684.x 15683381 10.1111/j.0001-6349.2005.00684.x

[15] Musa J, Daru PH, Mutihir JJ, Ujar IA Ectopic pregnancy in Jos Northern Nigeria: Prevalence and impact on subsequent fertility. Niger J Med. 2009;18:35–38. 19485145

[16] De Waard L, Butt JL, Muller CJ, Cluver Retrospective review of the medical management of ectopic pregnancies with methotrexate at a South African tertiary hospital. S Afr J OG. 2014;20(3):84–87. https://doi.org/10.7196/SAJOG.920

[17] Snyman LC, Makulana T, Makin JD A randomised trial comparing laparoscopy with laparotomy in the management of women with ruptured ectopic pregnancy. SAMJ. 2017;107(3):258–263. https://doi.org/10.7196/SAMJ.2017.v107i3.11447 28281433 10.7196/SAMJ.2017.v107i3.11447

[18] Amoko D, Buga G Clinical presentation of ectopic pregnancy in Transkei, South Africa. East Afr Med J. 1995;72(12):770–773. 8689974

[19] Prasanna B, Jhansi CB, Swathi K, Shaik MV A study on risk factors and clinical presentation of ectopic pregnancy in women attending a tertiary care centre. Int Archiv Int Med. 2016;3(1):90–96.

[20] Constant D, Grossman D, Lince N, Harries J Self-induction of abortion among women accessing second-trimester abortion services in the public sector, Western Cape Province, South Africa: An exploratory study. S Afr Med J. 2014 Apr;104(4):302–305. 25118559 10.7196/samj.7408

[21] Harries J1, Cooper D, Strebel A, Colvin CJ Conscientious objection and its impact on abortion service provision in South Africa: A qualitative study. Reprod Health. 2014 Feb 26;11(1):16 https://doi.org/10.1186/1742-4755-11-16 24571633 10.1186/1742-4755-11-16 PMC3996040

[22] Statistics South Africa. [homepage on internet] City of Tshwane [cited 2017 Oct 14]. Available from: http://www.statssa.gov.za/?page_id=1021&id=city-of-tshwane-municipality

[23] Vaquez G, Winston RM, Brosens IA Tubal mucosa and ectopic pregnancy. J Obstet Gynaecol. 1983;90:468–474. 10.1111/j.1471-0528.1983.tb08946.x 6849848

